# Feasibility of Ti-6Al-4V Alloys with Nanoporous and Nanotubular Surfaces for the Treatment of Femoral Defects

**DOI:** 10.3390/ijms27104335

**Published:** 2026-05-13

**Authors:** Daniel Alves dos Santos, Guilherme Arthur Longhitano, Gustavo Andrade Fraga, Rogerio Leone Buchaim, Daniela Vieira Buchaim, João Paulo Mardegan Issa, Vinicius Barroso Hirota, Marina Ribeiro Paulini, André Antonio Pelegrine, Arnaldo Rodrigues Santos, Rui Seabra Ferreira Junior, Marcelo Rodrigues da Cunha

**Affiliations:** 1 Postgraduate Program in Health Sciences, Faculty of Medicine of Jundiaí (FMJ), Jundiaí 13202-550, Brazil; daniel.santos@ceunsp.edu.br (D.A.d.S.); drgustavofraga1@gmail.com (G.A.F.); 2 Centro de Tecnologia da Informação Renato Archer, Campinas 13069-901, Brazil; guilonghita@gmail.com; 3 Graduate Program in Anatomy of Domestic and Wild Animals, Faculty of Veterinary Medicine and Animal Science, University of Sao Paulo (FMVZ/USP), Sao Paulo 05508-270, Brazil; rogerio@fob.usp.br (R.L.B.); danibuchaim@alumni.usp.br (D.V.B.); 4 Department of Biological Sciences, Bauru School of Dentistry (FOB/USP), University of Sao Paulo, Bauru 17012-901, Brazil; 5 Medical School, University Center of Adamantina (FAI), Adamantina 17800-000, Brazil; 6 Department of Postgraduate, Dentistry School, Faculty of the Midwest Paulista (FACOP), Piratininga 17499-010, Brazil; 7 Department of Basic and Oral Biology, School of Dentistry of Ribeirao Preto, University of Sao Paulo, Ribeirao Preto 14040-900, Brazil; jpmissa@forp.usp.br (J.P.M.I.); marina.paulini@usp.br (M.R.P.); 8 Fatec de Esportes, Centro Paula Souza, São Paulo 02180-021, Brazil; vbhirota@gmail.com; 9 Department of Implant Dentistry, Faculdade São Leopoldo Mandic, Campinas 13045-755, Brazil; andre.pelegrine@slmandic.edu.br; 10 Centro de Ciências Naturais e Humanas (CCNH), Universidade Federal do ABC (UFABC), São Bernardo do Campo 09606-070, Brazil; arnaldo.santos@ufabc.edu.br; 11 Botucatu Medical School (FMB), São Paulo State University (UNESP), Botucatu 18618-687, Brazil; rui.seabra@unesp.br; 12 Center for the Study of Venoms and Venomous Animals (CEVAP), São Paulo State University (UNESP—Universidade Estadual Paulista), Botucatu 18610-307, Brazil; 13 Center for Translational Science and Biopharmaceutical Development (CTS-CEVAP), São Paulo State University (UNESP—Universidade Estadual Paulista), Botucatu 18610-307, Brazil

**Keywords:** implants, titanium, additive manufacturing, bone regeneration, osseointegration, bone repair, FEG-SEM, nanoporous, nanotubular

## Abstract

Treating fractures involving complex bone defects remains a major challenge in regenerative medicine. Additive manufacturing enables the fabrication of patient-specific implants, while surface anodization may enhance osseointegration by improving cell adhesion at the bone–implant interface. This study evaluated the feasibility of porous Ti-6Al-4V implants produced by additive manufacturing and surface-modified by anodization for repairing critical-sized femoral defects. Eighty rats were assigned to four groups: control (no graft), non-anodized Ti-6Al-4V implants, nanoporous implants, and nanotubular implants. After six weeks, bone regeneration and mechanical performance were assessed. Newly formed bone volume was significantly higher in the implanted groups compared to control (24.62 ± 3.11%), reaching 47.25 ± 3.92% (non-anodized), 58.20 ± 5.39% (nanoporous), and 40.72 ± 2.22% (nanotubular). Bone strength was also greater in implanted groups (150.27 ± 2.94 N, 151.98 ± 10.37 N, and 156.59 ± 4.95 N, respectively) compared to control (128.16 ± 2.17 N), with no significant differences among treated groups. No signs of implant rejection or inflammation were observed, indicating good biocompatibility. Anodized surfaces demonstrated enhanced osteogenic performance, particularly in the nanoporous group. These findings indicate that anodized porous Ti-6Al-4V implants produced by additive manufacturing combine biological compatibility with mechanical stability, supporting their potential application in bone reconstruction therapies.

## 1. Introduction

Musculoskeletal injuries of the lower limbs are severe and rank among the most prevalent clinical conditions worldwide, with substantial functional, social, and economic impacts [1]. Their main causes include osteosarcomas, osteomyelitis, high-energy trauma (particularly motor vehicle accidents), osteodegenerative diseases, sports-related overload, and age-associated conditions such as osteoporosis [2,3]. The consequences of these injuries are broad and often disabling, encompassing bone mass loss, avascular necrosis of soft and hard tissues, deformities, osteoarthritis, functional impairment, claudication, amputations, and complications such as compartment syndrome, fat embolism, infections, and deep vein thrombosis [4,5,6]. Many of these complications are particularly common in inadequately treated fractures, which increase morbidity rates and hospital costs, especially in femoral fractures due to their therapeutic complexity [7,8,9].

Femoral injuries alone affect more than one million individuals worldwide each year [10,11]. In such cases, selecting an appropriate therapeutic approach is essential to ensure timely recovery while reducing hospitalization time and the risk of osteoarticular, vascular, and respiratory complications [12]. Accordingly, osteosynthesis has become one of the most commonly used methods for treating femoral fractures, as it relies on implants that stabilize the fracture and promote bone consolidation [13,14,15].

Ti-6Al-4V alloys are widely used in clinical practice due to their favorable mechanical properties [16]. However, their relatively high elastic modulus may induce bone resorption at the recipient site and impair osseointegration [17,18]. Therefore, continued technological innovation remains necessary, as the orthopedic indication of these materials depends on fatigue resistance, toughness, stiffness, and the ability of the resulting implants to promote effective bone–implant interaction. In this context, persistent challenges in the treatment of complex bone defects have driven advances in manufacturing techniques, particularly additive manufacturing. This approach enables the development of patient-specific implants tailored to the geometry and severity of bone defects [19]. Its clinical feasibility was demonstrated by Jardini et al. [20], who fabricated a three-dimensional titanium alloy biomodel successfully used in cranial trauma reconstruction.

Additive manufacturing integrates data from computed tomography and magnetic resonance imaging to map bone lesions and fabricate patient-specific three-dimensional models that can serve as bone substitutes [21]. This process also promotes the formation of more homogeneous microstructures with a lower incidence of structural imperfections [22]. In addition, additive manufacturing allows control over the chemical composition and surface topography, improving material stability and corrosion resistance [23]. As a result, metallic implants produced by additive manufacturing combine high mechanical strength with relatively low weight, thus contributing to patient comfort [24].

Additive manufacturing relies on layer-by-layer fabrication methods, enabling the production of complex metallic structures [18,21]. Among metal additive manufacturing technologies, such as Direct Energy Deposition (DED), Binder Jetting, and Powder Bed Fusion (PBF), PBF is currently the most developed and widely used technique for producing orthopedic implants. It enables the fabrication of complex porous structures with controlled pore size, reducing stress shielding and promoting vascularized bone ingrowth [25,26]. Despite these advantages, surfaces obtained by additive manufacturing still present limitations, such as surface roughness and imperfections, which may compromise cell adhesion after implantation into host tissues. Moreover, implant surfaces often exhibit limited osteogenic activity due to their bioinert nature [27].

To overcome these limitations, several surface modification techniques have been proposed. For instance, hydroxyapatite coatings improve bioactivity and osteoconductivity, while physical vapor deposition (PVD) allows the formation of thin and uniform coatings. However, these techniques may present drawbacks such as coating delamination, poor long-term stability, or cracking under mechanical stress [28].

In contrast, electrochemical anodization produces a titanium dioxide (TiO_2_) layer organized into nanotubular or nanoporous structures, enhancing surface energy and hydrophilicity, and thereby promoting cellular adhesion and proliferation. Additionally, anodization can be applied to complex three-dimensional geometries, such as porous scaffolds, without compromising structural integrity [29].

Anodization is an electrochemical surface treatment that modifies both the topography and physicochemical properties of materials [30,31,32]. In addition to altering surface architecture, anodization enhances implant resistance to corrosion, wear, and fatigue, thus extending their durability [33]. Importantly, this technique enables the formation of nanotubular and nanoporous features that favor cellular proliferation and biological integration [30,31,32].

From a clinical perspective, combining additive manufacturing with anodization yields implants with high osseointegration potential, reduced inflammatory response, and increased functional longevity. This approach is particularly promising for applications in anatomically critical regions such as the spine, hip, knee, and craniofacial structures [34]. Accordingly, these manufacturing and surface-modification strategies may help address clinical challenges such as bone resorption and susceptibility to corrosion, both of which compromise implant osseointegration. In this context, the aim of this study was to investigate whether surface anodization enhances the osteogenic performance of porous Ti-6Al-4V implants manufactured by additive manufacturing, by comparing non-anodized implants with anodized implants presenting nanoporous or nanotubular surface topographies in a femoral defect model.

## 2. Results

### 2.1. Metallic Implants Characterization

**Figure 1:**
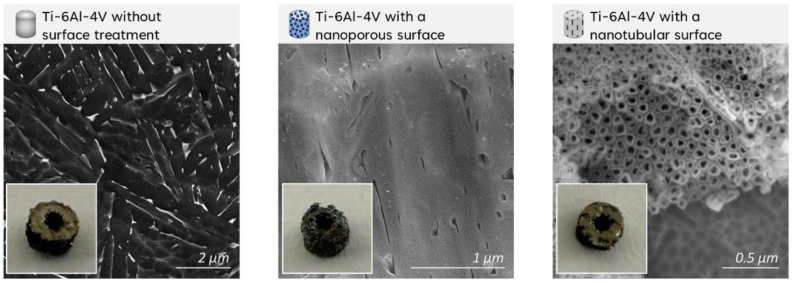
FEG-SEM images of the surface topographies of uncoated, nanoporous, and nanotubular Ti-6Al-4V implants, as well as the macroscopic appearance of the implants after surface modification.

Figure 1 shows the FEG-SEM images of the Ti-6Al-4V implant surfaces without surface treatment and after anodization with nanoporous and nanotubular topographies. The untreated surfaces presented a typical Widmanstätten (basket-weave) microstructure, characterized by parallel lamellae of the α/α′ phases and β-phase crystals (white phase) nucleated between the lamellae. During electron beam melting, the electron beam interacts with the powder bed forming a localized melt pool, which undergoes extremely rapid cooling due to its small volume. This process promotes the formation of a refined martensitic microstructure. During fabrication of subsequent layers, the powder bed is maintained at approximately 600 °C [35], which promotes partial decomposition of martensitic α′ and precipitation of β phase crystals while remaining below the β-transus temperature of the alloy (≈995 °C).

**Figure 2:**
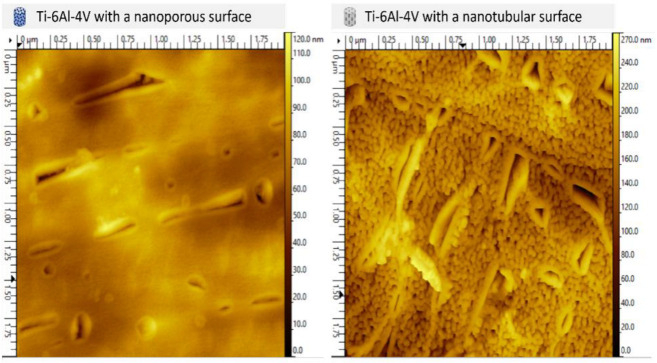
AFM topographic maps of the nanoporous and nanotubular Ti-6Al-4V implants.

After anodization, the samples exhibited visible changes in surface appearance, displaying different colors depending on the thickness of the oxide layer formed, as shown in the inserts of Figure 1. Atomic force microscopy maps are shown in Figure 2. Under the 20 V–5 min condition, the top layer consisted predominantly of nanopores, whereas under the 20 V–60 min condition the surface exhibited both nanopores and nanotubes. The nanopores and nanotubes measured diameters were 11.4 ± 2.9 and 45.1 ± 10.5 nm, respectively. As a result of the difference in nanostructures’ diameters, the surfaces presented roughness values of Sa = 14.0 ± 5.4 nm and Sq = 17.2 ± 6.4 nm for nanopores, and of Sa = 28.2 ± 2.9 nm and Sq = 36.6 ± 4.0 nm for nanotubes. Contributing to increasing the roughness values, surface heterogeneities arising from compositional differences between the α/α′ phase and the β phase were observed. The β phase contains a higher concentration of vanadium, a β-stabilizing element in titanium alloys [30].

The oxide thickness was 126.9 ± 9.5 nm for the nanoporous oxide and 835.0 ± 48.9 nm for the nanotubular oxide. However, despite the differences in oxide morphology and thickness, the amount of fluorine incorporated into the surfaces detected by energy-dispersive spectroscopy (EDX) was 9.4 ± 0.3 at% for the nanoporous layer and 11.1 ± 0.9 at% for the nanotubular layer, as shown in Table 1. According to the literature, anodized samples doped with fluoride ions are associated with antibacterial activity [32,36,37].

Finally, Table 2 presents the contact angle and surface free energy (γFowkes) for the uncoated, nanoporous, and nanotubular surfaces. All the surfaces presented contact angles < 90°, indicating hydrophilic behavior. When comparing the results, uncoated and nanoporous surfaces presented similar surface free energy values, whereas the nanotubular surface presented the lowest contact angle and, thus, the highest surface free energy values. This result can be related to the high nanometric roughness of the nanotubular topography.

### 2.2. Macroscopic and Radiological Analysis of the Surgical Area

**Figure 3:**
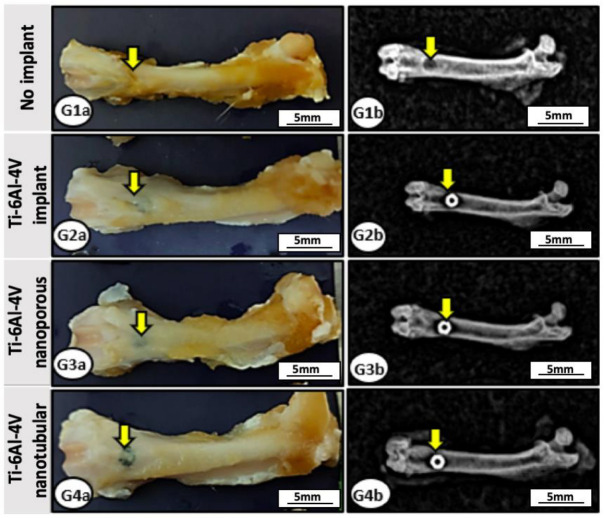
Macroscopic and radiographic evaluation of the operated femur. Representative macroscopic (G1a–G4a) and radiographic (G1b–G4b) images of the operated femur from animals in the experimental groups (G1–G4). Overall anatomical integrity of the femur and preservation of the surgical site (arrows) are observed, with no macroscopic or radiographic signs of tissue damage. In the implanted groups (G2–G4), the implants are visible and properly positioned within the bone defect. G1, control (no implant); G2, Ti-6Al-4V implant without surface treatment; G3, Ti-6Al-4V implant with a nanoporous surface; G4, Ti-6Al-4V implant with a nanotubular surface.

In all experimental groups (G1, control no implant; G2, Ti-6Al-4V implant without surface treatment; G3 Ti-6Al-4V implant with a nanoporous surface; G4 Ti-6Al-4V implant with a nanotubular surface), no macroscopic alterations were observed in the surgical site, including signs of inflammation, changes in the coloration of soft or bone tissues indicative of tissue necrosis, bone deformities, secondary fractures, or pseudoarthrosis (Figure 3).

Radiological evaluation showed that complete regeneration of the bone defect did not occur; however, femoral architecture remained preserved, with clearly defined radiopaque cortical bone and no evidence of bone rarefaction. In G1, the center of the bone defect exhibited a radiolucent image, whereas in the implanted groups, radiodensity corresponding to the metallic implants was observed. The implants remained properly fixed within the bone defect (Figure 3).

### 2.3. Histological Analysis of Bone Repair

In all groups, no inflammatory infiltrates were observed at the surgical site, including multinucleated giant cells, macrophages, lymphocytes, or neutrophils. New bone formation originated from the margins of the bone defect and exhibited immature and lamellar characteristics, consistent with an ongoing bone remodeling process. However, this newly formed bone did not completely close the bone defects.

**Figure 4:**
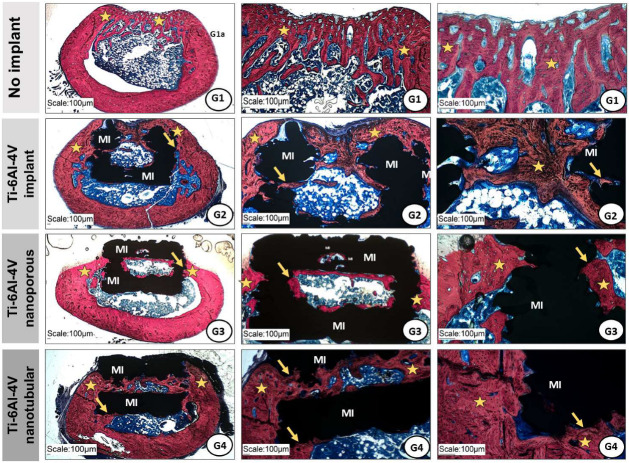
Representative photomicrographs of the surgical area of the femur from animals in the experimental groups (G1–G4) stained with Stevens Blue. Bone neoformation (asterisks) originates from the margins of the bone defect in all groups. In the implanted groups (G2–G4), the metallic implants (MI) are surrounded by newly formed bone undergoing osseointegration (arrows), as indicated by the absence of intervening connective tissue at the bone–implant interface.

In the control group (G1), bone formation at the lesion site appeared thinner and more trabecular. In contrast, in the implanted groups (G2–G4), bone growth was denser and occurred along the internal and external surfaces of the metallic implants, without interposition of connective tissue, indicating osseointegration (Figure 4).

**Figure 5:**
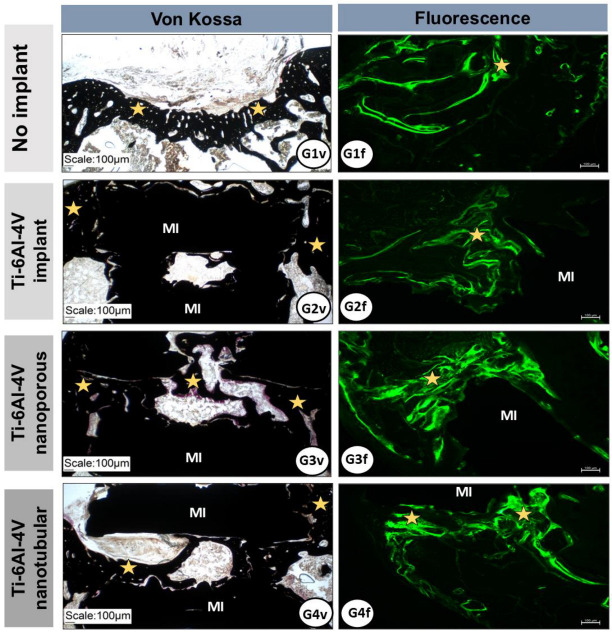
Representative photomicrographs of the femoral surgical area from animals in the experimental groups (G1–G4) stained using Von Kossa and fluorescence techniques. Von Kossa staining (G1v–G4v) demonstrates mineralization of the newly formed bone (yellow asterisks) in all groups. Fluorescence microscopy (G1f–G4f) highlights newly formed bone tissue, visualized in green. MI, metallic implant.

Additionally, isolated areas of new bone formation were observed within the fenestrations of the metallic implants. Von Kossa staining indicated mineralization of the bone formed in the surgical area, and fluorescence labeling marked the bone growth process in all experimental groups (Figure 5).

### 2.4. Histomorphometric Evaluation of Newly Formed Bone Volume and Biomechanical Analysis

The percentage of newly formed bone volume was 24.62 ± 3.11 in G1, 47.25 ± 3.92 in G2, 58.20 ± 5.39 in G3, and 40.72 ± 2.22 in G4. Newly formed bone volume differed significantly among groups (*p* < 0.05), except between G2 and G4, which showed similar values. Among the groups, G3 exhibited the highest amount of newly formed bone.

Regarding bone strength in the surgical area, the values (Newton, N) for groups G1 to G4 were 128.16 ± 2.17 N, 150.27 ± 2.94 N, 151.98 ± 10.37 N, and 156.59 ± 4.95 N, respectively. Statistical analysis indicated that all comparisons involving G1 (G1 vs. G2, G1 vs. G3, and G1 vs. G4) were significantly different (*p* < 0.05), whereas no statistically significant differences were observed among the implanted groups (G2 vs. G3, G2 vs. G4, and G3 vs. G4) (Table 3).

Values represent mean ± standard deviation. Statistical significance (*p* < 0.05): Newly formed bone volume differed significantly among groups except between G2 and G4; G3 showed the highest volume. Bone strength comparisons showed significant differences for all G1 vs. G2/G3/G4, but no differences among G2, G3, and G4.

## 3. Discussion

Partial or total repair or replacement of compromised bone tissue remains technically and clinically challenging. For this reason, alternative bioengineering strategies have become increasingly important to address this condition and other clinical challenges, such as implant rejection and poor osseointegration of metallic devices used for fracture stabilization [38]. In studies involving bone tissue regenerative medicine, it is important to evaluate the macroscopic and histological responses of bone neoformation following stimulation by a biomaterial [39,40,41]. The success of bone implants depends on both material biocompatibility and effective osseointegration [42].

The macroscopic findings of the present study are consistent with previously reported data for additively manufactured titanium implants [43,44]. Animals in the implanted groups (G2 to G4) showed good clinical recovery, with no signs of infection, fibrosis, implant rejection, necrosis, deformities, secondary fractures, or pseudoarthrosis. The absence of postoperative complications, together with appropriate healing of adjacent soft tissues, further supports the biocompatibility of anodized implants, a critical requirement for orthopedic clinical applications [39,40,41].

The satisfactory biocompatibility observed in this study can be attributed to several factors, including proper implant sterilization prior to implantation [43], appropriate pre- and postoperative animal care [41,45], acceptance of the implant by host bone tissue due to titanium quality, and the absence of secondary fractures, implant migration, or deformation, indicating adequate mechanical resilience. In addition, anodization-induced nanotopography has been shown to reduce inflammatory responses and the risk of implant rejection [46], possibly due to increased wettability, surface energy, and chemical stability of TiO_2_, features commonly associated with nanostructured surfaces. Based on this macroscopic evidence supporting implant quality, the next stage of validation involved radiological assessment of fracture repair and implant osseointegration at the surgical site [47].

Radiographic analyses revealed partial bone repair of the femoral defect after six weeks in the implanted groups, with preservation of cortical margins and absence of bone rarefaction, osteomyelitis, or pseudoarthrosis. In the control group, a radiolucent area persisted at the center of the lesion due to the predominance of connective tissue with low radiodensity, whereas the implanted groups exhibited radiodensity compatible with metallic implants and initial bone filling [48]. Implant stability at the surgical site was also confirmed, as no displacement or deformation was observed. Continuous abrasion between implants and host bone may release metallic particles, triggering inflammatory reactions and reducing implant bioactivity and longevity [49]. However, such complications were not observed in the present study.

The macroscopic and radiological findings meet the initial criteria for biomaterial use in reconstructive surgeries and justify further histological and biomechanical analyses [43]. The clinical indication of bone substitutes depends not only on the absence of toxicity and immunogenicity but also on their ability to promote a microenvironment favorable to osteogenesis [50]. Therefore, evaluation of the implant capacity to stimulate bone growth is essential [51,52].

Histological results demonstrated that, in control group (G1), the bone defect remained predominantly filled with connective tissue. In contrast, implanted groups showed progressive bone formation from the defect margins toward the lesion center, with direct bone–implant contact and small foci of neoformation within implant fenestrations, indicating effective migration and integration with host bone. The bone volume formed in the implanted groups exceeded that of the control group, demonstrating the osteogenic potential of the evaluated surfaces. Failures in bone–implant integration are among the main causes of clinical failure [53,54], a condition not observed in the present experimental model.

Nanostructured surfaces obtained by anodization favored implant fixation within the femoral recipient sites due to the formation of an organized TiO_2_ layer in the form of nanotubes or nanopores, which increases contact area, wettability, and surface energy [55]. These characteristics enhance cell adhesion, osteoblastic differentiation, and matrix mineralization [52,56,57]. The osteogenic effects of anodized implant surfaces were further confirmed through fluorescence and Von Kossa staining. The presence of nanotubes and nanopores considerably increases bone–implant contact area, accelerating mineral matrix deposition and subsequent osseointegration [58,59].

Anodization enables the formation of ordered nanotubular TiO_2_ structures, enhancing surface bioactivity [60]. Other studies have also reported improved cell adhesion and proliferation on TiO_2_ nanotubular surfaces compared with smooth scaffolds due to increased hydrophilicity [61,62]. Based on these findings, greater bone volume formation was expected in G4, which received nanotubular implants. However, superior osteogenic performance was observed in G3, which received nanoporous implants. Although nanotubular surfaces are widely described as highly bioactive and hydrophilic, the present results suggest that, under the conditions evaluated, nanoporous topography provided a more favorable microenvironment for bone formation. This may be attributed to the greater surface homogeneity of nanoporous structures, which enhances protein adsorption and initial cell adhesion—key events in osteogenesis. In contrast, the inferior performance observed in the nanotubular group may be associated with specific features of its nanoarchitecture. The average nanotube diameter (45.1 ± 10.5 nm) is below the range considered optimal (~70 nm) for osteogenic gene expression and enhanced osseointegration [63]. Additionally, parameters such as nanotube length, organization, and nanoscale roughness are known to directly influence cell adhesion, proliferation, and differentiation, potentially limiting the biological response when not properly optimized. Nevertheless, both nanotubular and nanoporous implants are promising, as they exhibited greater bone volume formation than the control group [64]. Despite these differences in bone formation, no significant differences in mechanical strength were observed among the implanted groups. This may indicate that a plateau in early biomechanical stability was reached, in which initial fixation and partial bone ingrowth are sufficient to support load-bearing conditions. It is important to emphasize that the biomechanical results obtained in this study represent the mechanical behavior of the bone—implant construct rather than the intrinsic properties of the bone tissue alone, since the compressive test was performed directly on the defect region containing the implant, thereby assessing the combined structural integrity of the regenerated bone and the implanted material. In this context, mechanical performance appears to be more strongly influenced by scaffold macroarchitecture—such as porosity and structural design, than by surface nanotopography alone. Therefore, although nanoporous surfaces demonstrated enhanced osteogenic performance, this biological advantage may not immediately translate into measurable mechanical gains during the early stages of healing [63,65].

Pore size, porosity, and interconnectivity facilitate implant osseointegration [66]. Complementing these advantages, porous titanium alloy scaffolds fabricated by 3D printing enhance osteoblast adhesion and proliferation [67]. Porous titanium scaffolds with approximately 60% porosity exhibit favorable mechanical properties and good cytocompatibility, supporting their potential for clinical application [68].

Scaffold porosity enhances cell proliferation, which is essential for bone growth [69,70,71,72]. However, concerns remain regarding mechanical resistance relative to the quantity and quality of bone formed in response to porous stimulation [68]. Porous titanium alloy design is a viable strategy to reduce elastic modulus and material stress [73]. Porous implants also facilitate fluid circulation, nutrient and blood exchange, oxygen transport, and vascularization, thereby promoting new bone formation [74]. Conversely, mechanical resistance decreases progressively with increasing porosity [75,76]. In the present study, biomechanical resistance values among implanted groups were similar and higher than those of the control group.

Although reduced mechanical strength with increasing porosity has been reported [75,76], the balance observed in this study suggests that additive manufacturing combined with anodization provides controlled porosity sufficient to stimulate osteogenesis without compromising structural stability. This manufacturing approach allows adjustment of elastic modulus in porous structures to match human bone stiffness, improving biomechanical compatibility while increasing the surface area available for bone growth [77].

Processes such as powder bed fusion enable modulation of implant elastic modulus and stiffness to approximate the properties of cancellous bone, thereby reducing stress shielding and promoting balanced regeneration [77,78]. Biomechanical similarity among implanted groups further supports the suitability of both nanostructured surface types for bone reconstruction.

The convergence of macroscopic, radiological, histological, and biomechanical findings demonstrates that nanoporous and nanotubular implants are favorable for bone repair surgeries. These outcomes included absence of inflammatory infiltrates [79], mechanical resistance superior to the control group, direct bone–implant contact without intervening connective tissue [80], bone neoformation along implant surfaces [77], absence of micromovements or implant deformation [81,82], absence of structural failures in implants or surrounding bone tissue [83] and greater bone volume formation compared with the control group due to the osteogenic effects of anodization. Titanium surfaces fabricated by powder bed fusion have also been reported to stimulate osteoblastic differentiation [83,84,85]. Collectively, these findings confirm that nanostructural surface modification enhances osseointegration, which is essential for satisfactory implant performance in the reconstruction of femoral lesions.

Although nanotubular surfaces are commonly described as highly conducive to cell adhesion and osteogenic activity, the present investigation demonstrated greater bone neoformation in the nanoporous group. This outcome may be explained by variations in nanotube diameter, surface uniformity, and the initial adsorption of proteins involved in cell attachment and differentiation. In this context, the findings are in agreement with previous reports showing that the biological performance of anodized titanium is strongly influenced by nanostructural geometry and anodization parameters [86,87].

This study presents some limitations that should be considered when interpreting the findings. First, the experimental model involved a single species, defect site, and follow-up period of six weeks, which may not fully reproduce the biological and mechanical complexity of segmental femoral defects in humans. In addition, the use of a non-load-bearing model represents a further limitation when extrapolating the results to clinical conditions. Second, only one implant geometry and specific anodization parameters were evaluated, preventing a broader understanding of how different pore architectures, nanostructure dimensions, and loading conditions might influence osseointegration and long-term stability. In this context, it is important to highlight the need to investigate implants with nanopores and nanotubes of different diameters, as the literature still shows divergence regarding the ideal size. However, only a single diameter was evaluated due to ethical limitations related to the number of animals used, in accordance with ARRIVE guidelines and NC3Rs principles [88]. Furthermore, the absence of quantitative microbiological, molecular, and gene expression analyses limits conclusions regarding the antibacterial performance and osteoimmunomodulatory effects of the anodized surfaces. Future studies should explore longer follow-up periods, larger animal models, and optimized scaffold designs, as well as combine nanoporous and nanotubular topographies with bioactive ions or drug-loading strategies to further enhance bone regeneration and translation to clinical applications.

## 4. Materials and Methods

### 4.1. Fabrication of Ti-6Al-4V Implants

**Figure 6:**
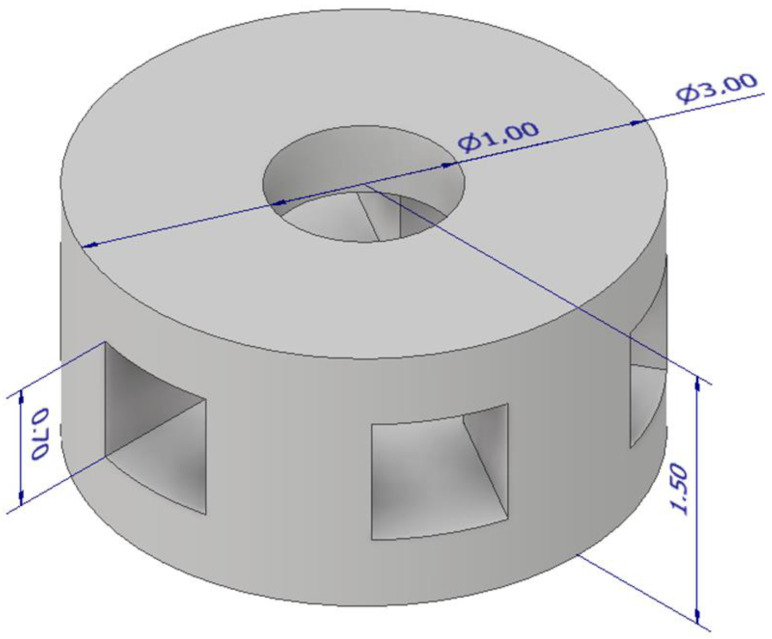
Porous Ti-6Al-4V implant fabricated by electron beam powder bed fusion, showing the overall geometry and dimensions of the implant (dimensions in mm).

Ti-6Al-4V implants were fabricated by electron beam powder bed fusion using a Q10 Plus system (GE Additive). The manufacturing parameters were based on previously reported protocols [30,89]. The implants were produced as cylindrical discs measuring 3 mm in diameter and 1.5 mm in height (Figure 6), with interconnected porosity designed to reduce stiffness and mass while enabling vascularized and mineralized tissue ingrowth [90,91].

Additive manufacturing processes based on powder bed fusion often leave unmelted or partially melted powder particles adhered to the surface of the fabricated components [31,90]. When such materials are intended for biomedical implantation, the potential release of powder particles into the bloodstream must be considered [31,92,93].

To remove residual powder particles, the implants underwent chemical polishing after fabrication. This method is particularly effective for cleaning internal surfaces of complex geometries such as interconnected pores [94]. The samples were immersed for 120 s in a solution containing HF (40%), HNO_3_ (65%), and H_2_O at a volumetric ratio of 1:4:5, under agitation at 20 ± 2 °C.

### 4.2. Microstructural Characterization and Surface Functionalization

For microstructural analysis, samples were ground and polished using 0.04 μm colloidal silica. Chemical etching was performed with Kroll’s reagent (5 vol% HNO_3_ and 10 vol% HF in H_2_O) for 25 s.

Implant surfaces were subsequently functionalized by electrochemical anodization following previously described protocols [30,65]. A programmable power supply (Hikari HF-3205 P) was used to control applied voltage. The electrolyte consisted of 1 M NH_4_H_2_PO_4_ and 0.3 M NH_4_F, adjusted to pH 4.4 and maintained at 20 ± 2 °C. A platinum cathode (12 cm^2^) served as the counter electrode.

Nanostructured surfaces were produced using a constant voltage of 20 V, applying anodization times of 5 min (nanoporous surface) and 60 min (nanotubular surface).

Surface morphology and chemical composition were analyzed using a field-emission gun scanning electron microscope (FEG-SEM MIRA 3 XMU, Tescan, Kohoutovice, Czech Republic) equipped with energy-dispersive X-ray spectroscopy (EDX). Three surface conditions were obtained: Non-anodized Ti-6Al-4V implants; Ti-6Al-4V implants with nanoporous surface; and, Ti-6Al-4V implants with nanotubular surface.

To measure the thickness of the oxide layers, a scalpel was used to scratch the oxide. Then, cracked oxide layers were searched on the scratched surface by FEG-SEM to find cracked parts of the oxide. The nanostructured morphologies were measured using ImageJ software (version 1.54i). The nanostructured morphologies were measured using ImageJ software.

Topographic maps and area roughness (Sa and Sq) on functionalized surfaces were measured by atomic force microscopy (AFM) in a NanoPark NX10 microscope, Park Systems, Suwon, Republic of Korea. Inter-mittent contact mode with an FMR-W pointer (f = 75 kHz) was used in a 2 × 2 µm area. Data was measured using Gwyddion software (version 1.52).

Contact angle was measured on a Theta Attension CAM 200 from KSV Instruments, Helsinki, Finland. Measurements were conducted using deionized water (polar liquid) and diiodome-thane (apolar liquid) at room temperature and air as the gas phase. The drops consisted of 0.7 µL. The contact angles were calculated using Young-Laplace adjustments. Five measurements were made for each condition. The surface energy for all conditions was calculated using the Fowkes theory approach [95]:

$$\gamma_{Fowkes}=\;\gamma_{s}^{D}+\gamma_{s}^{P}\;[\mathrm{ergs}/{\mathrm{cm}}^{2}]$$

where $\gamma_{s}^{D}$ is the dispersive and $\gamma_{s}^{P}$ is the polar forces components of the solid substrate, which relate by:

$$\frac{(1+cos\;cos\;\theta \;)\gamma_{l}}{2\sqrt{\gamma_{l}^{D}}}=\sqrt{\frac{\gamma_{s}^{P}\gamma_{s}^{D}}{\gamma_{l}^{D}}}+\sqrt{\gamma_{s}^{D}}$$

where *θ* is the measured contact angle between the solid–liquid interface, $\gamma_{l}$ is the liquid surface free-energy and $\gamma_{l}^{D}$ is the dispersive forces component of the liquid surface free-energy. For diiodomethane, $\gamma_{l}^{D}=50.8\;ergs/cm^{2}$ and $\gamma_{l}^{P}=0.0\;ergs/cm^{2}$, thus $\gamma_{l}=\gamma_{l}^{D}+\;\gamma_{l}^{P}=50.8\;ergs/cm^{2}.$ For water, $\gamma_{l}^{D}=21.8\;ergs/cm^{2}$ and $\gamma_{l}^{P}=51.0\;ergs/cm^{2}$, thus $\gamma_{l}=\gamma_{l}^{D}+\gamma_{l}^{P}=72.8\;ergs/cm^{2}$.

### 4.3. Implant Cleaning and Sterilization

Prior to implantation, the implants underwent standardized cleaning and sterilization protocol to ensure surface purity and reproducibility of the experimental conditions.

Initially, the implants were cleaned by ultrasonic treatment in a neutral detergent solution at 30–45 °C for 10–30 min to remove organic contaminants such as oils, residues, and debris. This step was followed by ultrasonic rinsing in high-purity deionized water, which promotes the removal of microscopic particles and soluble salts through cavitation effects.

Subsequently, the samples were thoroughly rinsed with fresh deionized water to eliminate any remaining detergent residues. Drying was performed using filtered compressed air or in a controlled-temperature oven to prevent airborne contamination. When required, a passivation treatment using citric or nitric acid was carried out to stabilize the implant surface and enhance corrosion resistance.

After the cleaning procedure, the implants were subjected to sterilization. Sterilization was performed by steam autoclaving at 121 °C under 15 psi pressure for 15–20 min, followed by a drying cycle to ensure complete removal of moisture. This method effectively eliminates bacteria, spores, and other microorganisms, ensuring sterility prior to use. In cases where heat-sensitive conditions were required, alternative sterilization methods such as gamma radiation were considered.

This combined cleaning and sterilization protocol ensures a contaminant-free and biologically safe surface. Previous studies have demonstrated that ultrasonic cleaning improves implant biocompatibility and reduces inflammatory responses [96,97], while proper sterilization is essential to prevent infection and ensure experimental reliability.

### 4.4. Experimental Design

All procedures were approved by the Animal Ethics Committee of the Faculty of Medicine of Jundiaí (CEUA/FMJ) under protocol no. 22322086. Eighty male Wistar rats (*Rattus norvegicus*), aged 15 weeks and weighing approximately 350 g, were used. Animals were obtained from the Institute of Energy and Nuclear Research (IPEN/USP) and housed in the animal facility of the Faculty of Medicine of Jundiaí. Animals were maintained in cages containing four individuals each, under enriched environmental conditions with ad libitum access to water and standard rat chow. The room temperature was maintained at 23 ± 1 °C with a 12 h light/dark cycle.

**Figure 7:**
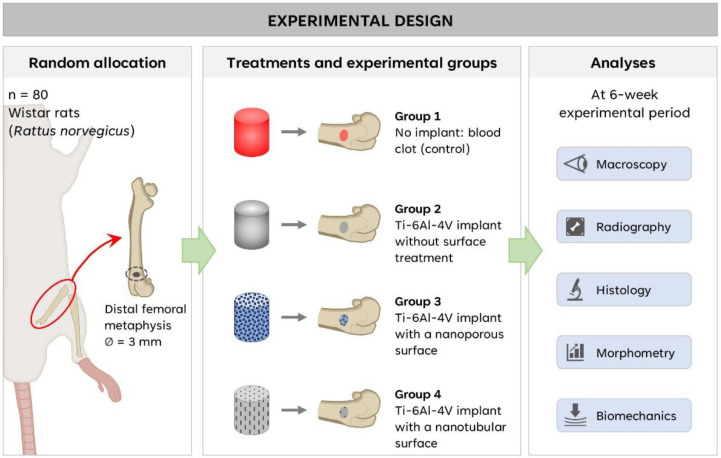
Experimental design and biomaterial characterization. A standardized 3-mm critical-size defect was created in the distal femur and animals were allocated into four experimental groups. Macroscopic, radiographic, histological, morphometric, and biomechanical analyses were performed after six weeks.

A standardized 3-mm critical-size bone defect was created in the distal metaphysis of the left femur. Animals were randomly allocated into four groups (*n* = 20 per group) (Figure 7): Group 1 (G1), defect filled with blood clot (control); Group 2 (G2), defect filled with non-anodized Ti-6Al-4V implant; Group 3 (G3), defect filled with nanoporous Ti-6Al-4V implant; Group 4 (G4), defect filled with nanotubular Ti-6Al-4V implant.

### 4.5. Surgical Procedure

Animals were anesthetized by intramuscular injection of ketamine and xylazine (1:1; 0.1 mL/100 g body weight). Postoperative analgesia was provided using tramadol hydrochloride (0.01 mg/100 g body weight) [43,98].

After anesthesia confirmation, animals were positioned in the supine position. The left thigh was shaved and disinfected with 2% chlorhexidine digluconate. A skin incision was performed followed by muscle dissection to expose the distal metaphysis of the femur. The periosteum was removed and a 3-mm surgical drill coupled to a low-speed handpiece was used to create the bone defect reaching the medullary canal. Continuous saline irrigation was applied to avoid overheating.

After implant placement, soft tissues were repositioned and sutured. During the first postoperative week, animals received daily intramuscular injections of pentabiotic for small animals (0.1 mg/100 g body weight; Fort Dodge^®^, Campinas, Brazil). Meloxicam was administered subcutaneously as an anti-inflammatory agent, and tramadol hydrochloride (0.01 mg/100 g body weight) was used for analgesia. Rifamycin spray (Rifocina^®^, Sanofi-Aventis Pharmaceutical Ltd., São Paulo, Brazil) was applied topically to the surgical site. In addition, dipyrone diluted in drinking water was provided during the postoperative period. Animals were continuously monitored to assess behavior and general health [43].

Six weeks after surgery, animals were euthanized in a quiet and private environment within the animal facility using thiopental sodium at a dose of 150 mg/kg (Thiopentax^®^, Cristália, Itapira, Brazil), administered intraperitoneally in the lower left abdominal quadrant, in combination with lidocaine hydrochloride (10 mg/kg; Xylestesin^®^, Cristália, Itapira, Brazil). After confirmation of death, the left femurs were removed, and samples from the surgical area were analyzed macroscopically, histologically, and mechanically.

### 4.6. Macroscopic and Radiological Analysis

The femurs were photographed using a Nikon D3500 DSLR digital camera, NIKON CORPORATION, Tokyo, Japan. Radiographic analysis was performed using an Odel system (300 mA, 40 kV, exposure time 0.06 s). Images were digitized using an Agfa^®^ imaging system.

### 4.7. Histological Analysis

Bone repair was evaluated using Stevens Blue, Von Kossa, and fluorescence staining.

Samples were sectioned transversely with an initial thickness of 200 μm, then ground to 30–50 μm using an Exakt Cutting-Grinding System. Stevens Blue staining was used to evaluate bone neoformation, while Von Kossa staining assessed mineralization [43].

For fluorescence analysis, animals received subcutaneous injections of Alizarin Red S (30 mg/kg; Sigma-Aldrich^®^, Merck KGaA, Darmstadt, Germany) on postoperative day 7 and calcein (10 mg/kg; Sigma-Aldrich™, Merck KGaA, Darmstadt, Germany) on postoperative days 14 and 21 to label the early stages of bone formation. Histological sections were analyzed using a laser scanning confocal microscope (TCS SP5 AOBS, Leica^®^, Wetzlar, Germany) coupled to a DFC 310 FX camera and QWin 3.1 software (Leica^®^, Wetzlar, Germany). Excitation wavelengths were 488 nm for calcein and 543 nm for Alizarin Red.

### 4.8. Histomorphometric and Statistical Analysis

Stevens Blue–stained images were used to quantify newly formed bone volume using Motic Images Plus 2.0 software (Motic Digital Microscopy^®^, Kowloon, Hong Kong). The percentage of bone formation was calculated as the ratio between newly formed bone volume and the total area of the bone defect. Data were analyzed with one-way ANOVA, followed by Tukey’s post hoc test, using BioEstat 5.3^®^ software. Results are expressed as means ± standard deviations, with the significance level set at *p* < 0.05.

### 4.9. Biomechanical Testing

Biomechanical testing was performed using a universal testing machine (Instron EMIC 23-2S) equipped with a 1 kN load cell. A compressive load of 5 N was applied using a 3-mm cylindrical indenter at a crosshead speed of 2 mm/min, following previously described protocols [43].

## 5. Conclusions

This study aimed to evaluate whether electrochemical anodization enhances the osteogenic performance of porous Ti-6Al-4V implants fabricated by additive manufacturing, comparing non-anodized, nanoporous, and nanotubular surfaces in a critical-sized femoral defect model in rats. Addressing a key gap in the literature—the limited in vivo evidence on the combined effects of controlled porosity from powder bed fusion and anodic TiO_2_ nanostructures on bone regeneration and biomechanics—our findings demonstrate superior bone neoformation (up to 58% in nanoporous group) and mechanical strength in anodized implants compared to ungrafted controls.

Both surface modifications exhibited excellent biocompatibility, direct bone–implant contact without intervening fibrous tissue, and mechanical stability, highlighting the innovation of integrating patient-specific porous scaffolds with nanofunctionalization in a single experimental platform. These results position anodized additively manufactured Ti-6Al-4V implants as promising candidates for femoral reconstruction, paving the way for clinical translation through further optimization of pore architecture, anodization parameters, and long-term evaluations.

## Figures and Tables

**Table 1:** EDX analysis of elemental composition for uncoated, nanoporous, and nanotubular Ti-6Al-4V implants.

Surface	Ti (at%)	Al (at%)	V (at%)	O (at%)	F (at%)
Uncoated	89.0 ± 4.9	7.0 ± 0.4	4.0 ± 0.1	-	-
Nanoporous	46.7 ± 0.8	5.4 ± 0.1	1.9 ± 0.1	36.6 ± 0.9	9.4 ± 0.3
Nanotubular	29.0 ± 2.9	3.2 ± 0.2	1.2 ± 0.1	55.5 ± 2.3	11.1 ± 0.9

**Table 2:** Contact angle and surface free energy measurements for uncoated, nanoporous, and nanotubular Ti-6Al-4V implants.

Surface	θ_H2O_ (°)	θ_diiodomethane_ (°)	γ_fowkes_ (ergs/cm^2^)
Uncoated	63.0	43.7	46.0
Nanoporous	26.1	21.5	51.2
Nanotubular	12.9	9.2	71.0

**Table 3:** Histomorphometric evaluation of newly formed bone volume and biomechanical analysis of bone strength across experimental groups.

Group	Newly Formed Bone Volume (%)	Bone Strength (N)
G1	24.62 ± 3.11	128.16 ± 2.17
G2	47.25 ± 3.92	150.27 ± 2.94
G3	58.20 ± 5.39	151.98 ± 10.37
G4	40.72 ± 2.22	156.59 ± 4.95

## Data Availability

Data presented in this study are available on request from the corresponding author.
